# Mitochondrial Transcription Factor A Deficiency in T Cells Leads to Activation of the Cyclic Guanosine Monophosphate–Adenosine Monophosphate Synthase/Stimulator of Interferon Genes Pathway and Production of Autoantibodies in Mice

**DOI:** 10.1155/jimr/2594317

**Published:** 2026-07-12

**Authors:** Shuhei Mashimo, Taku Kuwabara, Kei Ito, Tetuo Mikami, Akira Ishiko, Motonari Kondo

**Affiliations:** ^1^ Department of Molecular Immunology, Toho University School of Medicine, Ota-Ku, Tokyo, 143–8540, Japan, toho-u.ac.jp; ^2^ Department of Dermatology, Toho University School of Medicine, Ota-ku, Tokyo, 143–8540, Japan, toho-u.ac.jp; ^3^ Toho University Graduate School of Medicine, Ota-ku, Tokyo, 143–8540, Japan, toho-u.ac.jp; ^4^ Department of Pathology, Toho University School of Medicine, Ota-Ku, Tokyo, 143–8540, Japan, toho-u.ac.jp; ^5^ Department of Medical Technology, Faculty of Health Science, Tsukuba International University, Tsuchiura, Ibaraki, 300–0051, Japan, ktt.ac.jp

## Abstract

Mitochondrial transcription factor A (TFAM) is a key regulator of mitochondrial DNA transcription and replication. T cell‐specific TFAM‐deficient mice are immunocompromised, often succumbing to viral infection, and their T cells are unresponsive to T‐cell antigen receptor (TCR) stimulation, suggesting that TFAM‐mediated mitochondrial activity regulates T‐cell activity, such as effector function and memory formation. In contrast to the attenuation of immune response, TFAM deficiency may also induce inflammatory responses, raising the possibility that TFAM deficiency results in inflammation‐mediated autoimmune responses. Thus, besides regulating T‐cell function, TFAM plays important roles in autoimmune responses. However, its role in autoimmune diseases remains uncertain. We aimed to investigate the role of TFAM in autoimmune diseases using T cell‐specific TFAM‐deficient mice. We detected anti‐double‐strand (ds) DNA antibody, and interferon alpha (IFNα) and IFNγ in the serum of TFAM^fl/fl^ CD4Cre mice after 30 weeks of age. Mononuclear cell infiltration was observed in the kidneys. TFAM‐deficient T cells exhibited leakage of mitochondrial DNA into the cytoplasm. Cytoplasmic mitochondrial DNA was associated with activation of TANK‐binding kinase 1 (TBK1) and IFN regulatory factor 3 (IRF3)—the downstream molecules of the nucleic acid sensor cyclic guanosine monophosphate–adenosine monophosphate synthase/stimulator of IFN genes (cGAS/STING) machinery. mRNA expression of type I IFN genes was elevated in T cells from TFAM^fl/fl^ CD4Cre mice. The suppressive function of Foxp3^+^ regulatory T (Treg) cells, which play a major role in establishment of peripheral tolerance, was reduced in the absence of TFAM. Our findings suggest that T cells in TFAM‐deficient conditions may contribute to immune instability and autoimmune responses by inducing type I IFN‐mediated inflammatory responses.

## 1. Introduction

The binding of the T‐cell antigen receptor (TCR) to an antigenic peptide presented by the major histocompatibility complex (MHC) triggers a series of intracellular signaling cascades [1–3]. Within the first minute of T‐cell activation, lymphocyte‐specific protein kinase (Lck) initiates downstream signaling of the TCR by phosphorylating the immune receptor tyrosine‐based activation motifs (ITAMs) of the zeta chain of CD3 complexes [4, 5]. The phosphorylated ITAMs generate docking sites for the 70‐kDa zeta chain‐associated protein kinase (ZAP70). After binding to ITAMs, ZAP70 is activated by Lck; the activated ZAP70 phosphorylates the transmembrane protein linker for activation of T cells, the adaptor protein SH2 domain‐containing leukocyte protein 76 kDa (SLP76), and other signaling enzymes such as phospholipase C. The activities of these molecules propagate signaling events, such as an increase in the concentration of intracellular Ca^2+^ and activity of mitogen‐activated protein kinase (MAPK), also known as Ras‐MAPK and extracellular signal‐regulated kinase pathways [6]. Phosphorylation of proteins leads to the activation of other downstream signaling pathways, eventually inducing gene expression and effector functions [3, 7, 8].

In addition to the abovementioned phosphorylation mechanism, TCR signaling is supported by mitochondria located near the cytoplasmic region of the TCR [9, 10]. Mitochondria act as a signaling platform that coordinates T‐cell fate decisions involved in T‐cell activation and differentiation [10, 11]. These mitochondria provide cellular energy, ATP, that is essential for T‐cell function and metabolites that modulate gene expression through epigenetic modification, such as methylation and acetylation [11, 12]. These epigenetic modifications are closely related to the expression of cytokines and other effector genes in T cells. Recent studies have demonstrated that a decline in mitochondrial function in T cells triggers an immunological imbalance and immune disorders, such as autoimmune diseases [13]. Therefore, molecules regulating mitochondrial function might contribute to T‐cell activity [13, 14].

Mitochondrial proteins, regulating mitochondrial function, are encoded by mitochondrial and nuclear DNA [15]. Mitochondrial transcription factor A (TFAM) is a nuclear‐encoded mitochondrial protein required for DNA transcription, replication, and maintenance [15]. The conditional knockout of TFAM in hematopoietic cells in mice results in perinatal lethality owing to severe fetal anemia [16]. T cell‐specific deletion of TFAM by crossing TFAM‐floxed mice with CD4Cre transgenic mice leads to severe mitochondrial defects and lysosomal storage disorders [17]. Recent studies have revealed that TFAM deletion in T cells impairs antigen‐induced TCR responses and gene expression of effectors, such as cytokines [18]. Furthermore, deletion of TFAM in regulatory T (Treg) cells impairs suppressive functions, disrupts the maintenance of Treg cells in peripheral tissues, and induces tissue inflammation [19, 20], suggesting that TFAM deficiency can cause autoimmune diseases. TFAM is necessary for regulating T cell functions; however, the contribution of TFAM to autoimmune responses remains unclear.

In this study, we aimed to investigate how TFAM contributes to autoimmune symptoms using T cell‐specific TFAM‐deficient (TFAM^fl/fl^ CD4Cre) mice. Anti‐double‐strand (ds) DNA antibodies were detected in the serum of TFAM^fl/fl^ CD4Cre mice after 30 weeks of age but not in the serum of wild‐type mice. Inflammatory lesions in the kidneys and C3 accumulation in the glomeruli were observed in TFAM^fl/fl^ CD4Cre mice. The interferon regulatory factor 3 (IRF3) and TANK‐binding kinase 1 (TBK1) pathways may contribute to the spontaneous expression of type I interferon (IFN) in TFAM‐deficient T cells in association with mitochondrial DNA leakage. These findings suggest that activation of cytoplasmic nucleic acid sensors in TFAM‐deficient T cells might trigger the induction of proinflammatory responses and the production of autoantibodies in TFAM^fl/fl^ CD4Cre mice.

## 2. Materials and Methods

### 2.1. Mice

All mice were bred and maintained in a C57BL/6 background under specific pathogen–free conditions at the Toho University School of Medicine Animal Facility in accordance with the institutional guidelines [21, 22]. The body weight was assessed weekly. Toho University Animal Care and User Committee (No. 24‐581) and the Toho University Safety Committee for Recombinant DNA Experiments (No. 24‐587) approved the experimental procedures.

TFAM^fl/fl^ CD4Cre (TFAM^fl/fl^ × CD4Cre) mice were generated as previously described [18]. We first assessed Cre‐dependence of TFAM deletion. As shown in Figure S1, the levels of Tfam in CD4 T and CD8 T cells in TFAM^fl/fl^ CD4Cre mice were reduced. We also assessed the mitochondrial function in TFAM^fl/fl^ CD4Cre and littermate control mice. Oxygen consumption rates (OCRs) were measured using an FX HS mini flux analyzer (Seahorse Bioscience) in CD4 T cells from TFAM^fl/fl^ CD4Cre mice, which presented a low OCR. TFAM^fl/fl^ CD4Cre mice developed normally and showed a similar frequency of CD4 and CD8 single‐ and double‐positive thymocytes as their control littermates and C57BL/6 control mice, suggesting that Tfam is not required during early T‐cell development [14, 17]. T cell‐specific Tfam deficiency reduced the population of CD4 and CD8 T cells in the spleen of TFAM^fl/fl^ CD4Cre mice. These results indicated that Tfam deficiency is dependent on Cre expression. Therefore, we used C57BL/6 wild‐type mice as a control partner (referred to as “Control”) and compared them with TFAM^fl/fl^ CD4Cre mice to assess the TFAM function in T cells.

## 3. Detection of Autoantibodies Using Enzyme‐Linked Immunosorbent Assay (ELISA) and Indirect Immunofluorescence Assay

To measure autoantibodies, peripheral blood samples were collected from wild‐type and TFAM^fl/fl^ CD4Cre mice, and serum levels of anti‐dsDNA antibody were determined using ELISA kits (Alpha Diagnostics, San Antonio, TX, USA) according to the manufacturer’s instructions. Autoantibodies were detected through immunofluorescence using HEK293 cells as a substrate for autoantigen; the cells were seeded on cover slides. Sera from mice were diluted (1:100) in phosphate‐buffered saline (PBS) and incubated with the fixed HEK293 cell slides in a humidified chamber for 120 min at 25°C. Next, the slides were washed twice with PBS for 5 min, and antibody binding was detected with Dylight 488 conjugated goat anti‐mouse immunoglobulin G (IgG; 1:200; ROCKLAND, Limerick, PA, USA) for 120 min at RT. The nuclei were visualized using 4^′^, 6‐diamidino‐2‐phenylindole (DAPI) staining. The slides were then washed with PBS and mounted with coverslips for fluorescence microscopy using an Olympus BX63 microscope (Olympus Co., Tokyo, Japan).

### 3.1. Histology

The mice were intracardially perfused sequentially with saline containing heparin (Aventis, Paris, France) and 4% paraformaldehyde. Specimens were collected and processed using paraffin, as previously described [22, 23]. The kidney sections were stained with hematoxylin and eosin (HE) solution according to standard histological procedures [24]. Frozen kidney tissue was sectioned and subjected to immunofluorescence staining with fluorescein isothiocyanate (FITC)‐conjugated anti‐C3 antibody (1:100; Immunology Consultants Laboratory, Portland, OR, USA). The nuclei were visualized via DAPI staining.

### 3.2. Urinary Protein

Urinary protein levels were assessed using a test paper (Pretest 3aII; Wako Pure Chemical Industries, Tokyo, Japan) [22]. This colorimetric assay is specific for albumin and estimates urinary protein at the following five levels: (0) 0, (1) 30, (2) 100, (3) 300, and (4) 1000 mg/dL.

### 3.3. ELISA

The concentrations of IFNα, IFNγ, interleukin‐6 (IL‐6), and tumor necrosis factor‐α (TNFα) in serum were measured using the mouse IFNα ELISA kits (ThermoFisher Scientific, Waltham, MA, USA) and IFNγ, IL‐6, and TNFα kits (BD Biosciences, Becton Drive, Franklin Lakes, NJ, USA) according to the manufacturer’s instructions. Absorbance at 450 nm was measured using an iMark microplate reader (Bio‐Rad, Hercules, CA, USA), and the cytokine concentration was calculated using a standard curve generated with standard recombinant cytokines.

### 3.4. Flow Cytometry

Cells isolated from the spleen and lymph nodes were subjected to erythrocyte depletion through hypotonic lysis, incubated with a mixture of fluorescence‐conjugated antibodies at 4°C for 30 min, and subsequently washed three times with PBS containing 1% (v/v) fetal calf serum and 0.05% (w/v) sodium azide. CD4^+^ T and CD8^+^ T cells were enriched using a FACSAria Fusion cell sorter (BD Biosciences). Data were analyzed using Tree Star FlowJo version 10.5.3 (FlowJo, BD Biosciences). Gating strategies used for the preparation of CD4 T cells, CD8 T cells, B cells, CD25^+^ CD4 T cells for the suppression assay, CD25^+^Foxp3^+^ CD4 T cells from the thymus and spleen, and CD25^−^ CD4 responder T cells are shown in Figure S2.

### 3.5. In Vitro Treg‐Cell Suppression Assay

CD25^−^ CD4^+^ conventional T (Tconv) and CD25 CD4^+^ Treg cells from the spleen were enriched via cell sorting. The gating strategy is presented in Figure S3. Responder Tconv cells (CD45.1, 2 × 10^5^ cells) were labeled with CFSE according to the manufacturer’s instructions (Thermo Fisher, Waltham) and cultured for 5 days with irradiated splenocytes (1 × 10^5^ cells: CD45.2) and anti‐CD3ε antibody in the presence or absence of CD25^+^ CD4 Treg cells (CD45.2) either from TFAM^fl/fl^ CD4Cre or control mice [25]. CFSE levels on Tconv cells were then measured by gating on CD45.1^+^ cells via flow cytometry.

### 3.6. Antibodies

Antibodies used for flow cytometry (CD4 [GK1.5], CD8 [53‐6.7], B220 [RA3‐6B2], CD45.1 [A20], CD45.2 [104], TCRβ [H57‐597], and CD95 SA367H8]) were purchased from BioLegend (San Diego, CA, USA). Anti‐Foxp3 (FJK‐16 s), anti‐GL7 (GL7), and anti‐CXCR5 (2G8) were procured from BD. Anti‐PD‐1 (J43) and anti‐t‐bet (eBio4B10) were obtained from Thermo Fisher Scientific. For immunoblot analysis, anti‐IRF3, anti‐phosphorylated IRF3, anti‐HSP70, anti‐Histone H3, anti‐cytochrome oxidase subunit IV (Cox IV), anti‐TBK1, anti‐phosphorylated TBK1, and anti‐nuclear factor‐κB (NF‐κB) p65 antibodies were purchased from Cell Signaling Technologies (Danvers, MA, USA).

### 3.7. Relative Quantification of Cytosolic Mitochondrial DNA

Cytosolic and mitochondrial cellular fractions of T cells (equal numbers of cells per sample) were prepared using the mitochondrial isolation kit for mammalian cells (ThermoFisher Scientific) according to the manufacturer’s instructions. Mitochondrial DNA from the cytosolic and mitochondrial fractions was isolated and purified using the DNeasy Blood and Tissue kit (Qiagen, Hilden, Germany). Mitochondrial DNA was quantified using quantitative polymerase chain reaction (PCR) with the primers Cox1_FW 5^′^‐gccccagatatagcattccc‐3^′^, Cox1_RV 5^′^‐gttcatcctgttcctgctcc‐3^′^, Nd1_FW 5^′^‐caaacacttattacaacccaagaaca‐3^′^, and Nd1_RV 5^′^‐tcatattatggctatgggtcagg‐3^′^ and normalized to the expression of the nuclear DNA 18S gene (primers 18SrDNA_FW 5^′^‐tagagggacaagtggcgttc‐3^′^; 18SrDNA_RV 5^′^‐cgctgagccagtcagtgt‐3^′^).

### 3.8. OCR

The OCR was measured using an XF HS Mini Extracellular Flux Analyzer (Agilent Technologies, Santa Clara, CA, USA). The culture medium was changed to Seahorse XF RPMI‐based medium. CD4 T cells (2 × 10^5^ cells) were preincubated at 37°C for 60 min in the absence of CO_2_. The OCR was measured under basal conditions and in response to 1 µM oligomycin, 2 µM FCCP, and 0.5 µM rotenone/antimycin A (Mito Stress Test kit).

### 3.9. Real‐Time PCR

Real‐time PCR was performed as previously described [26, 27]. Briefly, total mRNA was isolated from cells using EasyPrep RNA (Takara Bio, Otsu, Japan) according to the manufacturer’s instructions. RNA (500 ng/reaction) was reverse‐transcribed using a high‐capacity RNA‐to‐cDNA kit (Applied Biosystems, Foster City, CA, USA). For quantitative analysis, real‐time PCR was conducted using the TaqMan Gene Expression Assay kit (Applied Biosystems, Foster City, CA, USA) on an Applied Biosystems 7500 Fast system. The following primers were used: Mm04207507_gh for *Ifna*, Mm00439552_s1 for *Ifnb*, Mm01231591_m1 for *Batf2*, Mm01260550_g1 for *Ifi35*, and Mm02619580_g1 for *Actb; Actb* encoding β‐actin was used as an endogenous reference for normalization. Quantitative real‐time PCR experiments were performed in triplicate twice.

### 3.10. Immunoblotting

Immunoblotting was performed as previously described [26, 27]. Briefly, the cells were resuspended in extraction buffer (50 mM Tris‐HCL [pH 7.4], 1% [v/v] Triton X‐100, 450 mM NaCl, 1 mM EDTA, 1 mM dithiothreitol, 1 mM sodium pyrophosphate, 20 mM sodium fluoride, and 1 mM sodium orthovanadate) and proteinase inhibitor mix. Lysates were centrifuged at 12,000 × *g* for 10 min at 4°C. Protein concentrations in the supernatants were determined using the bicinchoninic acid (Thermo Fisher Scientific) protein assay. The supernatants were suspended in sodium dodecyl sulfate‐polyacrylamide gel electrophoresis (SDS–PAGE) sample buffer (50 mM Tris‐HCl [pH 6.8], 10% [v/v] glycerol, 2% [v/v] SDS, 0.05% [w/v] bromophenol blue, 2% [v/v] 2‐ME, 0.05 mM sodium orthovanadate, and 0.5 mM EDTA) and boiled. They were then loaded onto 7.5% (w/v) or 10% (w/v) SDS–PAGE gels for protein separation. The resolved proteins were transferred onto polyvinylidene difluoride membranes in a transfer buffer (25 mM Tris, 0.192 M glycine, and 20% [v/v] methanol). The membranes were blocked with 1% (v/v) bovine serum albumin in Tris‐buffered saline (pH 7.5) containing 0.05% (v/v) Tween 20 at 25°C for 2 h, incubated with the indicated antibodies and anti‐mouse IgG or anti‐rabbit IgG coupled with horseradish peroxidase, and visualized using an enhanced chemiluminescence detection system (EZ‐Capture MG, ATTO, Tokyo, Japan). Quantitation was performed with ImageJ v. 1.49 software (National Institute of Health, Bethesda, MD, USA).

### 3.11. Statistical Analysis

Statistical analyses were performed using the mean difference hypothesis and the Student’s *t*‐test or, when the variances were unequal, the Mann–Whitney *U* test. A confidence level of 95% was adopted, with significance being *p*  < 0.05.

## 4. Results

### 4.1. TFAM^fl/fl^ CD4Cre Mice Produced Autoantibodies

**Figure 1:**
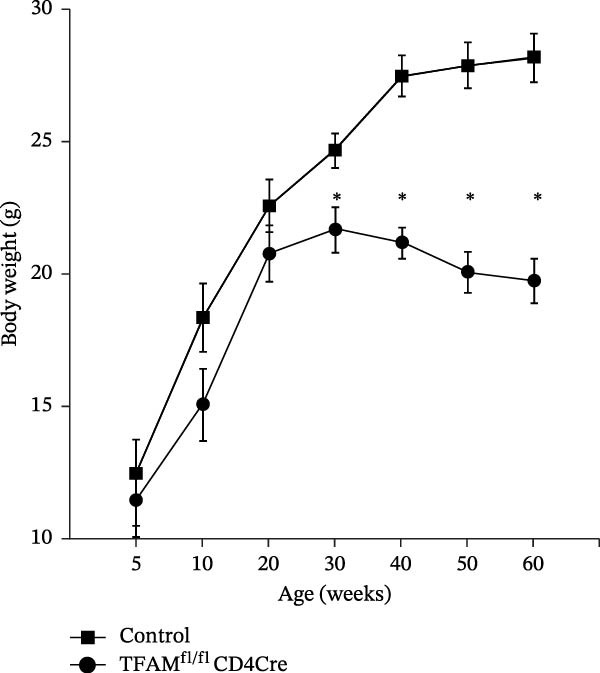
T cell‐specific deletion of TFAM resulted in lower body weight. T cell‐specific deletion of TFAM resulted in lower body weight. The body weight of each mouse was measured at the indicated period. Eight control (square) and eleven TFAM^fl/fl^ CD4Cre (circle) mice were used in each experimental group. The mean value ± SD is indicated. Statistical analysis was performed using the Student’s *t*‐test.  ^∗^ *p* < 0.01 compared with the control mice. TFAM, mitochondrial transcription factor A.

**Figure 2:**
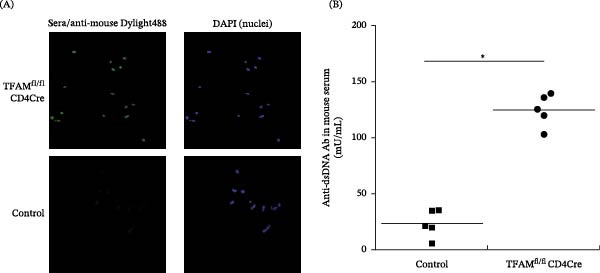
TFAM^fl/fl^ CD4Cre mice produced autoantibodies in serum. (A) Indirect fluorescence assay for detecting autoantibodies in serum from TFAM^fl/fl^ CD4Cre mice. HEK293 cells were probed with serum from control and TFAM^fl/fl^ CD4Cre mice (30 weeks of age), followed by Dylight488‐conjugated anti‐mouse Ig, and stained with DAPI. A representative result of more than three independent experiments is shown. (B) The amount of anti‐double‐strand (ds) DNA antibody in serum (30 weeks of age) from the control (square) and TFAM^fl/fl^ CD4Cre (circle) mice (assay range: 15.6–1000 mU/mL). Each symbol represents an individual mouse. Statistical analysis was performed using the Student’s *t*‐test.  ^∗^ *p* < 0.01 compared with the controls. *n* = 5. DAPI, 4′, 6′‐diamidino‐2‐phenylindole.

To define the intrinsic role of TFAM in T‐cells, we crossed mice carrying the loxP‐flanked TFAM allele with CD4Cre mice to generate TFAM^fl/fl^ CD4Cre mice. Compared with wild‐type control mice, TFAM^fl/fl^ CD4Cre mice showed a gradual reduction in body weight after 30 weeks of age (Figure 1). To further investigate immune dysfunction associated with TFAM deficiency, indirect immunofluorescence analysis for the detection of autoantibodies was performed. The substrate cells were cultured on glass coverslips overnight and fixed with cold methanol to permeate the cell membrane; diluted sera were applied to the coverslips. Indirect immunofluorescence analysis revealed nuclear staining in sera from TFAM^fl/fl^ CD4Cre mice, whereas no such staining was observed in sera from wild‐type mice (Figure 2A). Anti‐dsDNA antibody was detected in sera from mice after 30 weeks of age using ELISA (Figure 2B). These results suggest that auto‐reactive antibodies are spontaneously produced in TFAM^fl/fl^ CD4Cre mice.

### 4.2. TFAM^fl/fl^ CD4Cre Mice Develop Lupus‐Like Symptoms

**Figure 3:**
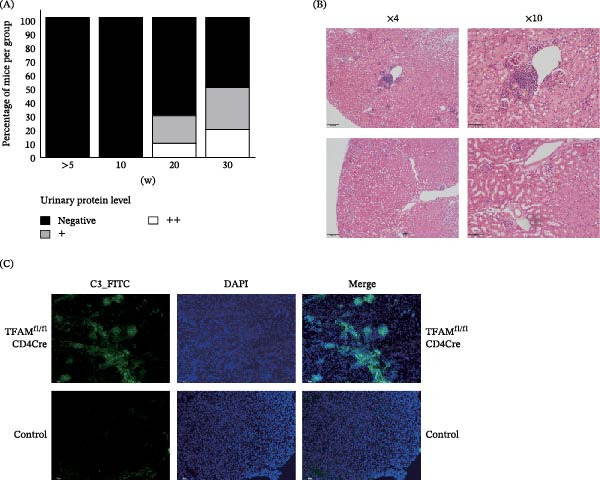
TFAM^fl/fl^ CD4Cre mice showed renal histopathology. (A) Frequency of TFAM^fl/fl^ CD4Cre mice (*n* = 10) with proteinuria at different weeks of age. The percentage of mice with detection levels of protein (+: 30–100; ++: 100–300 mg/dL) in urine is indicated. (B) Hematoxylin and eosin staining of renal sections from wild‐type (30 weeks) and TFAM^fl/fl^ CD4Cre (30 weeks) mice. Scale bar, 200 µm on the left and 100 µm on the right. (C) Sections of kidneys from control and TFAM^fl/fl^ CD4Cre mice stained with FITC‐conjugated anti‐mouse C3 and DAPI. Scale bar, 100 µm. DAPI, 4′, 6′‐diamidino‐2‐phenylindole; FITC, fluorescein isothiocyanate.

Increased levels of anti‐dsDNA antibodies imply systemic lupus erythematosus‐like disorders in TFAM^fl/fl^ CD4Cre mice. Hence, we tested urinary protein levels in TFAM^fl/fl^ CD4Cre mice and found that some of these mice, but none of the age‐matched wild‐type controls, tested positive for urinary protein after 20 weeks of age, and the number of protein‐positive mice increased by 30 weeks (Figure 3A). Increased urinary protein levels suggest renal lesions in TFAM^fl/fl^ CD4Cre mice. As expected, a considerable number of infiltrating mononuclear cells were observed in the perivascular regions of the kidneys (HE staining; Figure 3B). In addition, a component of complement C3 was detected around the glomerulus in the kidney (Figure 3C), suggesting that immune complexes were formed in TFAM^fl/fl^ CD4Cre mice. These results suggest that TFAM deletion triggers autoimmune responses and renal failure, leading to weight loss in TFAM^fl/fl^ CD4Cre mice.

**Figure 4:**
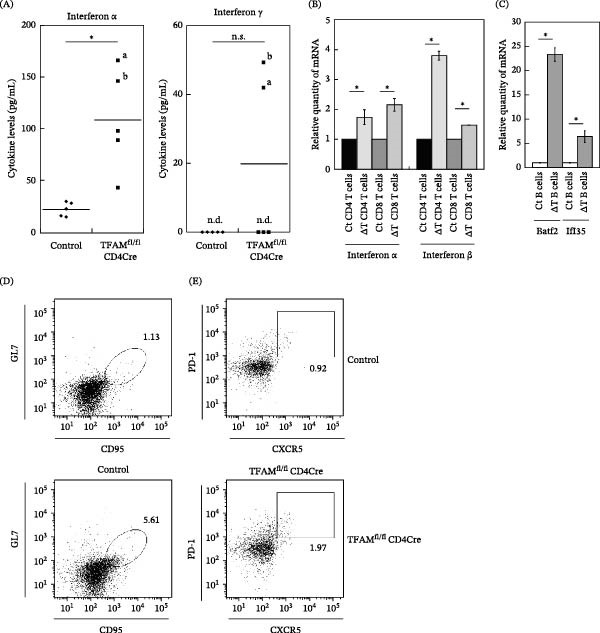
TFAM^fl/fl^ CD4Cre mice spontaneously expressed type I interferon. (A) Concentration of interferon alpha (IFNα, assay range: 31.3–2000 pg/mL) and IFNγ (assay range: 31.3–2000 pg/mL) in the serum of control and TFAM^fl/fl^ CD4Cre mice (30 weeks of age). Bars show the mean for *n* = 5 mice. Each symbol represents one mouse (control mice: 5; TFAM^fl/fl^ CD4Cre mice: 5). Symbols a and b indicate the same TFAM^fl/fl^ CD4Cre mice used for IFNα and IFNγ assays. (B) *IFNα* and *IFNβ* mRNA expression in CD4 T cells from the spleen of control (Ct) and TFAM^fl/fl^ CD4Cre (ΔT) mice. (C) *Batf2* and *Ifi35* mRNA expression in B220^+^ splenic B cells from control (Ct) and TFAM^fl/fl^ CD4Cre (ΔT) mice, determined via quantitative PCR. (D, E) Flow cytometry plots comparing GL‐7^+^ CD95^+^ cells in B220^+^ B cells and PD‐1^+^ CXCR5^+^ cells in CD4^+^ T cells from control and TFAM^fl/fl^ CD4Cre mice. Gating strategy is presented in Figure S3. Data for one experiment are representative of three independent experiments. Statistical analysis was performed using the Student’s *t*‐test.  ^∗^ *p* < 0.01 compared with the controls. n.s., not significant, *p*  > 0.05; n.d., not detected; Ct: control mice; ΔT: TFAM^fl/fl^ CD4Cre mice. Data are shown as mean ± SD. PCR, polymerase chain reaction; TFAM, mitochondrial transcription factor A.

We next analyzed serum cytokines in TFAM^fl/fl^ CD4Cre mice and their control littermates. Reflecting the observed autoimmune susceptibility, the levels of IFNα in the serum of 30‐week‐old TFAM^fl/fl^ CD4Cre mice were higher than those in control mice (Figure 4A, left, *n* = 5). Although serum IFNγ was not detected in three of the five TFAM^fl/fl^ CD4Cre mice, two mice with high IFNα levels also exhibited detectable serum IFNγ (Figure 4A, right). Despite the possibility of their involvement in the pathology of TFAM^fl/fl^ CD4Cre mice, we could not detect IL‐6 and TNF‐α in the serum of TFAM^fl/fl^ CD4Cre and control mice (data not shown).

We investigated whether CD4 T cells produce IFNs. T cells were sorted from the splenocytes of TFAM^fl/fl^CD4Cre and control mice (Figure S3), and the expression levels of IFNγ were assessed in freshly isolated CD4 T cells. IFNγ transcripts were not detected in CD4 T cells using quantitative PCR (data not shown). We further analyzed the expression of transcription factor t‐bet in CD4 T cells. CD4 T cells from TFAM^fl/fl^ CD4Cre mice did not express t‐bet (data not shown). In contrast to IFNγ, type I IFN transcripts showed spontaneous expression in CD4 T cells derived from TFAM^fl/fl^ CD4Cre mice (Figure 4B). TFAM‐deficient CD8 T cells also demonstrated upregulation of IFNα and IFNβ expression (Figure 4B).

Spontaneous cytokine production in TFAM^fl/fl^ CD4Cre mice suggested that type I IFN could stimulate B cells. To investigate this possibility, we assessed the expression of IFN‐stimulated genes using quantitative PCR. B cells from TFAM^fl/fl^ CD4Cre mice increased the mRNA expression of ISGs (*Batf2* and *IfI35*) (Figure 4C). Moreover, TFAM^fl/fl^ CD4Cre mice showed increased abundance of GL‐7^+^ CD95^+^ B and CXCR5^+^ PD‐1^+^ CD4 T cells in the spleen (Figure 4D,E). These findings suggest that type I IFNs may contribute to immune dysregulation in TFAM^fl/fl^ CD4Cre mice.

### 4.3. Mitochondrial DNA Leaks Into the Cytoplasm in TFAM‐Deficient T Cells

**Figure 5:**
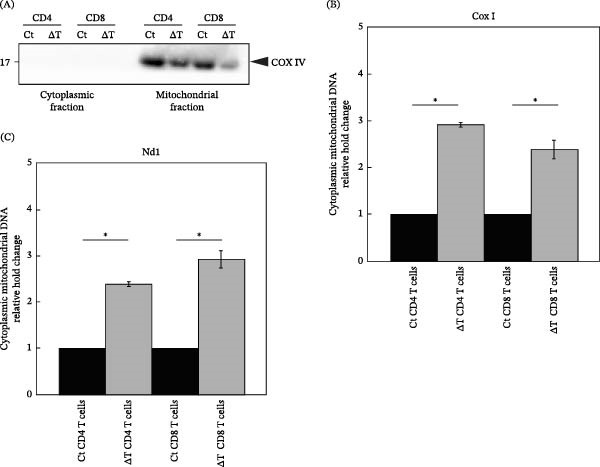
TFAM deficiency‐induced mitochondrial DNA release. T cells were prepared from the spleen of control and TFAM^fl/fl^ CD4Cre mice (30 weeks of age). Gating strategy is presented in Figure S3. (A) The levels of cytochrome oxidase subunit IV (COX IV) in the cytoplasmic and mitochondrial fractions prepared from these T cells (Ct: T cell from control mice and ΔT: T cells from TFAM^fl/fl^ CD4Cre mice) were assayed via immunoblot analysis. The data are representative of three similar experiments. (B, C) Quantitative PCR of cytoplasmic DNA prepared from control T cells (Ct) and TFAM^fl/fl^ CD4Cre T cells (ΔT) using primers for mitochondrial Cox I (B) and mitochondrial Nd1 (C) as mitochondrial genes. Statistical analysis was performed using the Student’s *t*‐test.  ^∗^ *p*  < 0.01 versus control T cells. Data are shown as mean ± SD. *n* = 3. Data for one experiment are representative of three independent experiments. PCR, polymerase chain reaction; TFAM, mitochondrial transcription factor A.

As TFAM contributes to maintaining the stability of mitochondrial DNA, we hypothesized that TFAM deficiency might result in mitochondrial instability, such as leakage of mitochondrial DNA into the cytoplasm. To determine the presence of mitochondrial DNA in the cytoplasm of TFAM‐deficient T cells, we purified naïve CD4 and CD8 T cells from control and TFAM^fl/fl^ CD4Cre mice (30 weeks of age) and prepared the cytoplasmic subcellular fraction from these T cells. Mitochondrial protein Cox IV was detected in the mitochondrial fraction but not in the cytoplasmic fraction (Figure 5A), suggesting that mitochondrial contamination was below the detection limit. The PCR analysis of two mitochondrially encoded genes, *Nd1* and *Cox I*, in the cytoplasmic fraction revealed the presence of cytoplasmic mitochondrial DNA specifically in TFAM^fl/fl^ CD4Cre T cells but not in wild‐type T cells, as observed using relative mitochondrial DNA quantification (Figure 5B,C). These results suggest that T cells lacking TFAM expression exhibited impaired mitochondrial DNA retention within the mitochondria.

### 4.4. Cytoplasmic Nucleic Acid Sensor Is Activated in TFAM‐Deficient T Cells

**Figure 6:**
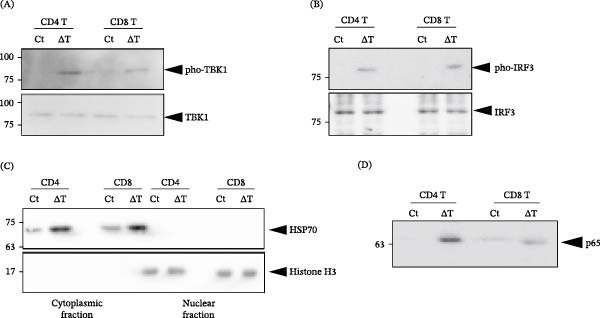
Mitochondrial DNA stimulates nucleic acid sensor machinery. T cells were prepared from the spleen of control and TFAM^fl/fl^ CD4Cre mice (30 weeks of age). Gating strategy is presented in Figure S3. Signaling molecule activation was examined in CD4 T and CD8 T cells from control (Ct) and TFAM^fl/fl^ CD4Cre (ΔT) mice. The cells were lysed, analyzed using SDS–PAGE, and immunoblotted with anti‐TBK1, anti‐phospho‐TBK1 (A), anti‐IRF3, anti‐phospho‐IRF3 (B), and anti‐NF‐κB p65 (D) antibodies. The levels of HSP70 and histone H3 in the cytoplasmic and nuclear fractions prepared from these T cells were assayed to confirm contamination of subcellular components in each fraction (C). Similar results were obtained for three independent experiments. Ct, control mice; ΔT, TFAM^fl/fl^ CD4Cre mice.

The release of mitochondrial DNA and mitochondrial dysfunction could synergistically induce the cyclic guanosine monophosphate–adenosine monophosphate synthase/stimulator of IFN genes (cGAS/STING) machinery as a sensor for cytoplasmic nucleic acid in T cells [28]. cGAS synthesizes cyclic guanosine monophosphate‐adenosine monophosphate (cGAMP). cGAMP binds to and activates the STING. The activated STING then recruits TBK1 to phosphorylate the transcription factor IRF3 and activate the NF‐κB pathway. IRF3 subsequently translocates to the nucleus along with NF‐κB to induce the expression of type 1 IFN and other target genes [29]. Thus, we investigated the activation of molecules of the cGAS/STING pathway in TFAM‐deficient T cells using immunoblot analysis. No significant change was observed in the expression levels of TBK1 and IRF3 in TFAM‐deficient T cells (both CD4 and CD8) compared with those in wild‐type T cells (Figure 6A,B); however, we observed the accumulation of phosphorylated TBK1 in TFAM‐deficient CD4 and CD8 T cells (Figure 6A). Furthermore, phosphorylated IRF3 was detected in TFAM‐deficient cells (Figure 6B). To investigate the contribution of NF‐κB in this pathway, we prepared nuclear fractions from T cells (Figure 6C). Consistently, nuclear p65 was detected in the nuclear fraction of TFAM‐deficient CD4 and CD8 T cells (Figure 6D), suggesting that IRF3 and NF‐κB were activated, inducing the transcription of their target genes in TFAM‐deficient T cells. These results suggest that TFAM‐deficient T cells experience mitochondrial stress, inducing the release of mitochondrial DNA into the cytoplasm. This subsequently triggers the nucleic acid sensor cGAS/STING pathway, potentially contributing to the development of type I IFN‐mediated inflammation in these mice.

### 4.5. T Cell‐Specific Deletion of TFAM Impairs the Suppressive Function of Treg Cells

**Figure 7:**
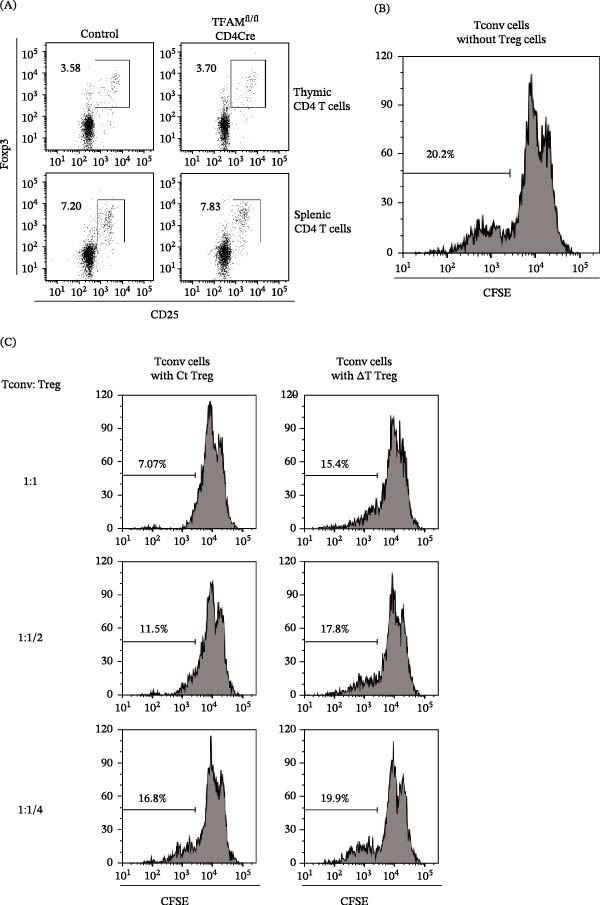
T cell‐specific TFAM deletion impairs the suppressive function of regulatory T cells. (A) Flow cytometric analysis of the thymic (top) and peripheral (spleen, bottom) Treg cells (30 weeks of age). CD25^+^ Foxp3^+^ CD4 T cells from control (left) and TFAM^fl/fl^ CD4 Cre (right) mice. The flow cytometry plots shown were pregated on the CD4‐positive fraction (Figure S2). (B, C) In vitro suppression assay with CFSE‐labeled Tconv cells (CD25^−^ CD4^+^) as responders and Treg cells derived from control (C, left) or TFAM^fl/fl^ CD4Cre (C, right) mice as suppressors. Results of cell division without Treg cells are shown in (B) as a control. Data for one experiment are representative of three independent experiments. CFSE, carboxyfluorescein succinimidyl ester.

Treg cells suppress autoimmune responses and play a central role in the establishment of peripheral immune homeostasis [30]. Treg cell deficiency and dysfunction may cause a T cell‐mediated autoimmune disorder [30]. Therefore, to determine the involvement of Treg cells in the autoimmunity of the TFAM^fl/fl^ CD4Cre mice, we first compared the frequency of thymic and splenic Treg cells. In TFAM^fl/fl^ CD4Cre mice at 28–30 weeks of age, the frequencies of CD4^+^ CD25^+^ Foxp3^+^ Treg cells in the thymus and periphery were comparable to those in control littermate mice (Figure 7A).

We next analyzed the suppressive function of Treg cells from TFAM^fl/fl^ CD4Cre mice in vitro. To determine whether TFAM‐deficient Treg cells suppress the proliferation of CD4^+^ CD25^−^ Tconv cells, we cultured activated CD4^+^ CD25^−^ T cells from CD45.1 congenic mice with or without CD4^+^ CD25^+^ Treg cells, most of which expressed Foxp3, from either TFAM^fl/fl^ CD4Cre or control littermate mice (CD45.2). After 5 days of culture, we analyzed the proliferation of the CD45.1 Tconv cells by CFSE dilution using a flow cytometer and observed the proliferation of Tconv cells (Figure 7B). Cell division in Tconv cells was clearly inhibited in the presence of Treg cells at ratios of 1:1 and 1:1/2 (Figure 7C, left). In contrast, Tconv cells proliferated even in the presence (1:1) of TFAM‐deficient Treg cells (Figure 7C, right). These results suggest that the function of Treg cells is impaired in TFAM^fl/fl^ CD4Cre mice.

## 5. Discussion

In the present study, we used the T cell‐specific TFAM‐deficient mice to investigate how TFAM contributes to autoimmune responses. Spontaneous production of the anti‐dsDNA antibody and infiltration of inflammatory mononuclear cells were detected in TFAM^fl/fl^ CD4Cre mice. We hypothesized that TFAM knockout in T cells results in susceptibility to autoimmune disease and analyzed T‐cell characteristics under mitochondrial defects. Mitochondrial DNA leaked from the mitochondria into the cytoplasm in TFAM‐deficient T cells. Serine/threonine TBK1, IRF3, and NF‐κB were activated by mitochondrial DNA in the cytoplasm of TFAM‐deficient T cells. TFAM‐deficient T cells also showed spontaneous expression of type I IFN, suggesting activation of the cGAS/STING pathway. Our results revealed that the loss of TFAM, accompanied by mitochondrial instability, triggers increased pro‐inflammatory T‐cell activity and results in susceptibility to autoimmune diseases.

TFAM, a mitochondrial protein encoded by the nuclear genome, regulates replication, transcription, and stability of mitochondrial DNA [31, 32]. The lack of TFAM in T cells results in severe mitochondrial DNA damage, mitochondrial respiratory chain deficiency, and reduced copy number of mitochondrial DNA [17, 18]. Consistent with the findings of these previous studies, we elucidated the role of mitochondria in T‐cell functions using T‐cell‐specific TFAM‐knockout mice [18]. The proliferation level of TFAM‐depleted T cells was less than that of wild‐type control T cells [17, 18]. Consistent with previous reports, T cells lacking TFAM showed substantially lower ZAP70 activity than the wild‐type T cells after cross‐linking the TCR with anti‐CD3 and anti‐CD28 antibodies [18]. These results suggest that T‐cell proliferation after TCR stimulation depends on the mitochondrial function. TFAM‐deficient T cells also exhibited below‐normal levels of mitochondrial reactive oxygen species [18], reduced generation of mitochondrial ATP, and lower activity of mitochondrial oxidative phosphorylation. Such mitochondrial damage and dysfunction are frequently observed in T cells from aged mice, suggesting that mitochondrial integrity is associated with T‐cell senescence [31]. Age‐associated changes in immunological functions include reduced production of IL‐2 and proliferation of T cells in older individuals [31]. The expression level of the TCR per cell does not change with aging; however, the functions of the antigen receptor might be impaired [32]. As the TCR repertoire of CD8 T cells used in response to repeated infection is significantly reduced by clonal expansion [33], T cells in older mice result in a skewed TCR repertoire and failure of total immune responses [34]. Therefore, T‐cell senescence impairs the efficiency of antiviral CD8 T‐cell responses [35, 36]. The helper function of CD4 T cells is also attenuated by senescence. In a previous study, upon transplantation of CD4 T cells from aged mice into young recipients, antigen‐specific proliferation of B cells and germinal center formation were considerably reduced after antigen immunization [37]. Recipients of aged CD4 donor cells also showed significantly decreased production of antigen‐specific immunoglobulin. The previous study also revealed that components essential for immunoglobulin production, such as B cells and follicular dendritic cells, do not exhibit age‐related decline in activities. In addition, the reduction in the activity of CD4 cells is not caused by a defect in the migration of donor T cells into the follicle but may be due to reduced expression of the co‐stimulatory molecule CD154, suggesting that senescent CD4 T cells fail to induce adequate function of B cells as a helper activity. Therefore, age‐related downregulation in T‐cell function contributes to defects in immune responses. In contrast, B‐cell expansion and immunoglobulin production were not impaired when CD4 donor cells from young mice were transferred into young or aged recipients [37, 38]. These results suggest that restoring senescent T cell function will improve immune responses, and the findings from analysis using TFAM^fl/fl^ CD4Cre mice may create new opportunities to address immunological senescence. We demonstrate that TFAM deficiency leads to mitochondrial dysfunction in the present study and reported attenuation of T‐cell activities in our previous study [18], suggesting that TFAM‐mediated mitochondrial damage accelerates the progression of premature senescence in T cells. The findings of this study also indicate that TFAM^fl/fl^ CD4Cre mice may serve as a valuable model for investigating immune senescence.

Our findings suggest that autoimmune responses occurred in TFAM^fl/fl^ CD4Cre mice, whereas previous studies have shown that T cells lacking TFAM expression exhibit reduced proliferation through TCR stimulation and TFAM mice with T cell‐specific deletion are immunocompromised [17, 18]. To address these contradictory results, we sought to clarify the importance of the TFAM function in autoimmune regulation. Mitochondrial DNA was detected in the cytoplasm of T cells, indicating that mitochondria could not retain DNA under the TFAM‐deficient conditions. Aberrant mitochondrial DNA in the cytoplasm of TFAM‐deficient T cells may be sensed through nucleic acid sensors, such as the cGAS/STING machinery, and activated T cells secrete an unexpected type I IFN protein. Moreover, a previous study demonstrated that type I IFN secreted by T cells induces lupus‐like autoimmunity in mice [39]. Therefore, the induction of anti‐dsDNA antibody production and C3 protein deposition in the kidneys are possible characteristics of TFAM^fl/fl^ CD4Cre mice. As disease symptoms triggered by the overproduction of type I IFN are believed to be wide, further investigation is necessary to define other indications of T cell‐mediated immune disorders. T cells, exposed to stress conditions such as chronic stimulation from persistent viral infection or infiltration into nutrient‐poor microenvironments, fail to maintain mitochondrial integrity. Under these conditions, mitochondria in T cells show reduced metabolism and mass and release mitochondrial DNA into the cytoplasm. Cytoplasmic mitochondrial DNA engages nucleic acid sensors and triggers type I IFN expression. Notably, some of the mechanistic details warrant clarification; however, the identification of mitochondrial DNA as a type I IFN‐inducible damage‐associated molecular pattern (DAMP) has important implications for gaining pathobiological insights into immune disorders.

T cells produce inflammatory cytokines and exhibit autoreactivity when mitochondrial damage or leakage of mitochondrial DNA occurs into their cytoplasm [40–42]. As a result, T cells subjected to mitochondrial dysfunction might induce symptoms similar to autoimmune diseases. The present study, together with previous studies [14, 17], demonstrated mitochondrial dysfunction in TFAM^fl/fl^ CD4Cre mice. Therefore, to assess the physiological consequences of TFAM deficiency in T cells, we analyzed changes in body weight and autoantibody production as indicators of impaired immune regulation. Consistent with previous studies showing that mitochondrial dysfunction in T cells leads to mitochondrial DNA leakage and inflammatory cytokine production [43], we demonstrated that TFAM‐deficient T cells spontaneously produced type I IFN and that TFAM^fl/fl^ CD4Cre mice produced anti‐dsDNA antibodies after 30 weeks of age. Senescence is considered a risk factor for autoimmunity [44]. Because TFAM^fl/fl^ CD4Cre mice exhibited premature senescence and mitochondrial dysfunction, they may be predisposed to autoimmune responses. However, a previous study reported that TFAM^fl/fl^ CD4Cre mice did not exhibit increased autoantibody production at 22 months of age, whereas age‐matched wild‐type mice showed elevated autoantibody levels [14]. Thus, despite the presence of premature senescence and mitochondrial dysfunction, TFAM^fl/fl^ CD4Cre mice were not reported to be autoimmune‐prone in the previous study. This discrepancy between the previous and present studies may be influenced by differences in breeding conditions and the commensal microbiota. The activity of TFAM‐deficient T cells may therefore be influenced by environmental factors.

We found that TFAM deficiency may contribute to autoimmune‐mediated renal failure. However, mitochondrial dysfunction induced by a lack of TFAM may also contribute to additional pathological conditions. Therefore, additional investigations are required to clarify the broader roles of mitochondria and TFAM in T cell function and autoimmune responses.

## 6. Conclusions

TFAM acts as a key regulator of transcription, replication, and maintenance of mitochondrial DNA. In this study, T cell‐specific TFAM‐knockout mice produced anti‐dsDNA antibodies and exhibited proteinuria after 30 weeks of age, whereas age‐matched wild‐type control mice did not exhibit proteinuria. As the mitochondria in TFAM‐deficient T cells fail to maintain mitochondrial integrity, signaling molecules associated with nucleic acid sensors appear to be activated, accompanied by the expression of type I IFNs in TFAM‐deficient T cells. These findings suggest that inflammatory responses associated with mitochondrial dysfunction may contribute to the pathogenesis of lupus‐like autoimmune pathology. To our knowledge, this study is among the first to describe a potential role of TFAM in T cell‐associated autoimmune diseases. TFAM^fl/fl^ CD4Cre mice may provide a useful model for investigating type I IFN‐associated autoimmune pathology and lupus‐like immune disorders.

## Funding

This work was supported by the Japan Society for the Promotion of Science Grants‐in‐Aid for Scientific Research (Grants 25460600, 17K08892, and 24K10264 to Taku Kuwabara) and the Toho University Project Research Grant (Grants 22‐22 and 23‐13 to Shuhei Mashimo).

## Conflicts of Interest

The authors declare no conflicts of interest.

## Supporting Information

Additional supporting information can be found online in the Supporting Information section.

## Supporting information


**Supporting Information** Figure S1. Analysis of TFAM deletion in T cells. (A) T cells were prepared from the spleen of control and TFAM^fl/fl^ CD4Cre mice. The gating strategy is presented in Figure 3. T cells were lysed, analyzed via SDS–PAGE, and immunoblotted with anti‐TFAM and anti‐β‐actin antibodies. (B) In the mitochondrial stress assay, changes in the oxygen consumption rate (OCR) were measured in real time after consecutive treatment with 1 μM of oligomycin, 2 µM FCCP, and 0.5 µM each of rotenone and antimycin A to induce mitochondrial stress. Mitochondrial activities of control and TFAM‐deficient CD4 T cells were analyzed using the Wave software (Seahorse Bioscience). (C) Flow cytometric plots showing CD4 and CD8 T‐cell population in thymocytes from control and TFAM^fl/fl^ CD4Cre mice. (D) Flow cytometric plots showing CD4 and CD8 T cells, and B220^+^ and TCRβ^+^ cells from the spleen. Gating strategies are presented in Figures S2 and S3. Representative results from more than three independent experiments are shown. Figure S2. Analysis of TFAM^fl/fl^ CD4Cre mice. Flow cytometric gating strategy used for the thymus. Figure S3. Analysis of TFAM^fl/fl^ CD4Cre mice. Flow cytometric gating strategy used for the spleen.

## Data Availability

The data that support the findings of this study are available from the corresponding author upon reasonable request.

## References

[1] Nel A. E. , T-Cell Activation through the Antigen Receptor. Part 1: Signaling Components, Signaling Pathways, and Signal Integration at the T-Cell Antigen Receptor Synapse, Journal of Allergy and Clinical Immunology. (2002) 109, no. 5, 758–770, 10.1067/mai.2002.124259. 11994696

[2] Nel A. E. and Slaughter N. , T-Cell Activation Through the Antigen Receptor. Part 2: Role of Signaling Cascades in T-Cell Differentiation, Anergy, Immune Senescence, and Development of Immunotherapy, Journal of Allergy and Clinical Immunology. (2002) 109, no. 6, 901–915, 10.1067/mai.2002.124965. 12063516

[3] Smith-Garvin J. E. , Koretzky G. A. , and Jordan M. S. , T Cell Activation, Annual Review of Immunology. (2009) 27, no. 1, 591–619, 10.1146/annurev.immunol.021908.132706. PMC2740335 19132916

[4] Molina T. J. , Kishihara K. , and Siderovski D. P. , et al.Profound Block in Thymocyte Development in Mice Lacking p56Lck, Nature. (1992) 357, no. 6374, 161–164, 10.1038/357161a0. 1579166

[5] Straus D. B. and Weiss A. , Genetic Evidence for the Involvement of the Lck Tyrosine Kinase in Signal Transduction Through the T Cell Antigen Receptor, Cell. (1992) 70, no. 4, 585–593, 10.1016/0092-8674(92)90428-F. 1505025

[6] van Leeuwen J. E. M. and Samelson L. E. , T Cell Antigen-Receptor Signal Transduction, Current Opinion in Immunology. (1999) 11, no. 3, 242–248, 10.1016/S0952-7915(99)80040-5. 10375551

[7] Courtney A. H. , Lo W.-L. , and Weiss A. , TCR Signaling: Mechanisms of Initiation and Propagation, Trends in Biochemical Sciences. (2018) 43, no. 2, 108–123, 10.1016/j.tibs.2017.11.008. 29269020 PMC5801066

[8] Gaud G. , Lesourne R. , and Love P. E. , Regulatory Mechanisms in T Cell Receptor Signalling, Nature Reviews Immunology. (2018) 18, no. 8, 485–497, 10.1038/s41577-018-0020-8. 29789755

[9] Baixauli F. , Martin-Cofreces N. B. , and Morlino G. , et al.The Mitochondrial Fission Factor Dynamin-Related Protein 1 modulates T-Cell Receptor Signalling at the Immune Synapse, The EMBO Journal. (2011) 30, no. 7, 1238–1250, 10.1038/emboj.2011.25. 21326213 PMC3094108

[10] Quintana A. , Pasche M. , and Junker C. , et al.Calcium Microdomains at the Immunological Synapse: How ORAI Channels, Mitochondria and Calcium Pumps Generate Local Calcium Signals for Efficient T-Cell Activation, The EMBO Journal. (2011) 30, no. 19, 3895–3912, 10.1038/emboj.2011.289. 21847095 PMC3209779

[11] Liu X. and Peng G. , Mitochondria Orchestrate T Cell Fate and Function, Nature Immunology. (2021) 22, no. 3, 276–278, 10.1038/s41590-020-00861-6. 33495653 PMC12416820

[12] Steinert E. M. , Vasan K. , and Chandel N. S. , Mitochondrial Metabolism Regulation of T Cell–Mediated Immunity, Annual Review of Immunology. (2021) 39, no. 1, 395–416, 10.1146/annurev-immunol-101819-082015. PMC10403253 33902315

[13] Desdin-Mico G. , Soto-Heredero G. , and Mittelbrunn M. , Mitochondrial Activity in T Cells, Mitochondrion. (2018) 41, 51–57, 10.1016/j.mito.2017.10.006. 29032101

[14] Desdin-Mico G. , Soto-Heredero G. , and Aranda J. F. , et al.T Cells with Dysfunctional Mitochondria Induce Multimorbidity and Premature Senescence, Science. (2020) 368, no. 6497, 1371–1376, 10.1126/science.aax0860. 32439659 PMC7616968

[15] Larsson N. G. , Wang J. , and Wilhelmsson H. , et al.Mitochondrial Transcription Factor a is Necessary for mtDNA Maintenance and Embryogenesis in Mice, Nature Genetics. (1998) 18, no. 3, 231–236, 10.1038/ng0398-231. 9500544

[16] Anso E. , Weinberg S. E. , and Diebold L. P. , et al.The Mitochondrial Respiratory Chain Is Essential for Haematopoietic Stem Cell Function, Nature Cell Biology. (2017) 19, no. 6, 614–625, 10.1038/ncb3529. 28504706 PMC5474760

[17] Baixauli F. , Acin-Perez R. , and Villarroya-Beltri C. , et al.Mitochondrial Respiration Controls Lysosomal Function during Inflammatory T Cell Responses, Cell Metabolism. (2015) 22, no. 3, 485–498. 26299452 10.1016/j.cmet.2015.07.020 PMC5026297

[18] Kuwabara T. , Ishikawa F. , and Ikeda M. , et al.SATB1-Dependent Mitochondrial ROS Production Controls TCR Signaling in CD4 T Cells, Life Science Alliance. (2021) 4, no. 11, 10.26508/lsa.202101093. PMC8500228 34583974

[19] Fu Z. , Ye J. , and Dean J. W. , et al.Requirement of Mitochondrial Transcription Factor a in Tissue-Resident Regulatory T Cell Maintenance and Function, Cell Reports. (2019) 28, no. 1, 159.e4–171.e4, 10.1016/j.celrep.2019.06.024. 31269437 PMC6679941

[20] Chapman N. M. , Zeng H. , and Nguyen T. M. , et al.mTOR Coordinates Transcriptional Programs and Mitochondrial Metabolism of Activated Treg Subsets to Protect Tissue Homeostasis, Nature Communications. (2018) 9, no. 1, 10.1038/s41467-018-04392-5. PMC5974344 29844370

[21] Kuwabara T. , Ishikawa F. , and Yasuda T. , et al.CCR7 Ligands are Required for Development of Experimental Autoimmune Encephalomyelitis Through Generating IL-23-Dependent Th17 Cells, The Journal of Immunology. (2009) 183, no. 4, 2513–2521, 10.4049/jimmunol.0800729. 19625643

[22] Fujita K. , Kuwabara T. , and Wang B. , et al.Irradiation Attenuates Systemic Lupus Erythematosus-Like Morbidity in NZBWF1 Mice: Focusing on CD180-Negative Cells, Journal of Immunology Research. (2023) 2023, 9969079. 37886369 10.1155/2023/9969079 PMC10599955

[23] Fujita K. , Akasaka Y. , and Kuwabara T. , et al.Pathogenesis of Lupus-Like Nephritis Through Autoimmune Antibody Produced by CD180-Negative B Lymphocytes in NZBWF1 Mouse, Immunology Letters. (2012) 144, no. 1-2, 1–6, 10.1016/j.imlet.2012.02.012. 22387632

[24] Akiba Y. , Kuwabara T. , Mukozu T. , Mikami T. , and Kondo M. , Special AT-Rich Sequence Binding Protein 1 is Required for Maintenance of T Cell Receptor Responsiveness and Development of Experimental Autoimmune Encephalomyelitis, Microbiology and Immunology. (2018) 62, no. 4, 255–268, 10.1111/1348-0421.12579. 29388727 PMC5947310

[25] Kondo M. , Tanaka Y. , Kuwabara T. , Naito T. , Kohwi-Shigematsu T. , and Watanabe A. , SATB1 Plays a Critical Role in Establishment of Immune Tolerance, The Journal of Immunology. (2016) 196, no. 2, 563–572, 10.4049/jimmunol.1501429. 26667169

[26] Matsui Y. , Kuwabara T. , Eguchi T. , Nakajima K. , and Kondo M. , Acetylation Regulates the MKK4-JNK Pathway in T Cell Receptor Signaling, Immunology Letters. (2018) 194, 21–28, 10.1016/j.imlet.2017.12.002. 29248490

[27] Kuwabara T. , Tanaka Y. , Ishikawa F. , Kondo M. , Sekiya H. , and Kakiuchi T. , CCR7 Ligands up-Regulate IL-23 through PI3-Kinase and NF-Kappa B Pathway in Dendritic Cells, Journal of Leukocyte Biology. (2012) 92, no. 2, 309–318, 10.1189/jlb.0811415. 22591694

[28] West A. P. and Shadel G. S. , Mitochondrial DNA in Innate Immune Responses and Inflammatory Pathology, Nature Reviews Immunology. (2017) 17, no. 6, 363–375, 10.1038/nri.2017.21. PMC7289178 28393922

[29] Cai X. , Chiu Y. H. , and Chen Z. J. , The cGAS-cGAMP-STING Pathway of Cytosolic DNA Sensing and Signaling, Molecular Cell. (2014) 54, no. 2, 289–296, 10.1016/j.molcel.2014.03.040. 24766893

[30] Sakaguchi S. , Wing K. , and Yamaguchi T. , Dynamics of Peripheral Tolerance and Immune Regulation Mediated by Treg, European Journal of Immunology. (2009) 39, no. 9, 2331–2336, 10.1002/eji.200939688. 19662638

[31] Escrig-Larena J. I. , Delgado-Pulido S. , and Mittelbrunn M. , Mitochondria during T Cell Aging, Semin Immunol 69, 2023. 10.1016/j.smim.2023.101808 37473558

[32] Martini H. and Passos J. F. , Passos, Cellular Senescence: All Roads Lead to Mitochondria, The FEBS Journal. (2023) 290, no. 5, 1186–1202, 10.1111/febs.16361. 35048548 PMC9296701

[33] Douziech N. , Seres I. , and Larbi A. , et al.Modulation of Human Lymphocyte Proliferative Response with Aging, Experimental Gerontology. (2002) 37, no. 2-3, 369–387, 10.1016/S0531-5565(01)00204-2. 11772524

[34] Fulop T. , Gagne D. , and Goulet A. C. , et al.Age-Related Impairment of p56Lck and ZAP-70 Activities in Human T Lymphocytes Activated Through the TcR/CD3 Complex, Experimental Gerontology. (1999) 34, no. 2, 197–216, 10.1016/S0531-5565(98)00061-8. 10363787

[35] Callahan J. E. , Kappler J. W. , and Marrack P. , Unexpected Expansions of CD8-Bearing Cells in Old Mice, The Journal of Immunology. (1993) 151, no. 12, 6657–6669, 10.4049/jimmunol.151.12.6657. 8258683

[36] Yager E. J. , Ahmed M. , Lanzer K. , Randall T. D. , Woodland D. L. , and Blackman M. A. , Age-Associated Decline in T Cell Repertoire Diversity Leads to Holes in the Repertoire and Impaired Immunity to Influenza Virus, The Journal of Experimental Medicine. (2008) 205, no. 3, 711–723, 10.1084/jem.20071140. 18332179 PMC2275391

[37] Messaoudi I. , Lemaoult J. , Guevara-Patino J. A. , Metzner B. M. , and Nikolich-Zugich J. , Age-Related CD8 T Cell Clonal Expansions Constrict CD8 T Cell Repertoire and Have the Potential to Impair Immune Defense, The Journal of Experimental Medicine. (2004) 200, no. 10, 1347–1358, 10.1084/jem.20040437. 15545358 PMC2211915

[38] Eaton S. M. , Burns E. M. , Kusser K. , Randall T. D. , and Haynes L. , Age-Related Defects in CD4 T Cell Cognate Helper Function Lead to Reductions in Humoral Responses, The Journal of Experimental Medicine. (2004) 200, no. 12, 1613–1622, 10.1084/jem.20041395. 15611289 PMC2211991

[39] Simpson S. R. , Rego S. L. , and Harvey S. E. , et al.T Cells Produce IFN-α in the TREX1 D18N Model of Lupus-like Autoimmunity, The Journal of Immunology. (2020) 204, no. 2, 348–359, 10.4049/jimmunol.1900220. 31826941 PMC6946867

[40] Ye J. , Fu J. , and Hou H. , et al.Cytoplasmic DNA Sensing Boosts CD4(+) T Cell Metabolism for Inflammatory Induction, Life Medicine. (2023) 2, no. 3, 10.1093/lifemedi/lnad021, lnad021. 39872301 PMC11749111

[41] Quintero-Gonzalez D. C. , Munoz-Urbano M. , and Vasquez G. , Mitochondria as a Key Player in Systemic Lupus Erythematosus, Autoimmunity. (2022) 55, no. 8, 497–505, 10.1080/08916934.2022.2112181. 35978536

[42] Zhao T. V. , Sato Y. , Goronzy J. J. , and Weyand C. M. , T-Cell Aging-Associated Phenotypes in Autoimmune Disease, Frontiers in Aging. (2022) 3, 10.3389/fragi.2022.867950, 867950. 35821833 PMC9261367

[43] Li Y. , Shen Y. , and Jin K. , et al.The DNA Repair Nuclease MRE11A Functions as a Mitochondrial Protector and Prevents T Cell Pyroptosis and Tissue Inflammation, Cell Metabolism. (2019) 30, no. 3, 477.ee6–492.e6, 10.1016/j.cmet.2019.06.016. 31327667 PMC7093039

[44] Liu Q. , Zheng Y. , Goronzy J. J. , and Weyand C. M. , T Cell Aging as a Risk Factor for Autoimmunity, Journal of Autoimmunity. (2023) 137, 10.1016/j.jaut.2022.102947, 102947. 36357240 PMC10164202

