# Supplementary figures and images for: Mitochondrial Transcription Factor A Deficiency in T Cells Leads to Activation of the Cyclic Guanosine Monophosphate–Adenosine Monophosphate Synthase/Stimulator of Interferon Genes Pathway and Production of Autoantibodies in Mice
Source: J Immunol Res. 2026 Jul 12;2026:2594317. doi: 10.1155/jimr/2594317 (PMC13358363; doi:10.1155/jimr/2594317)

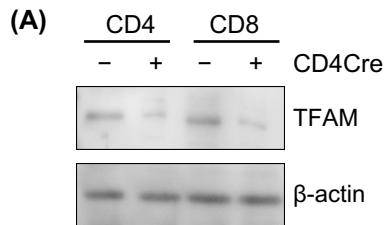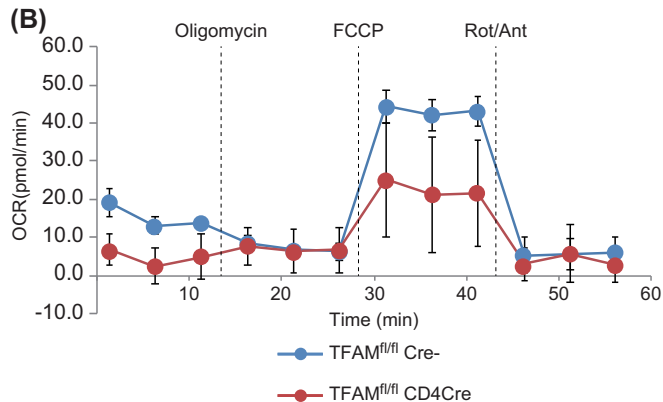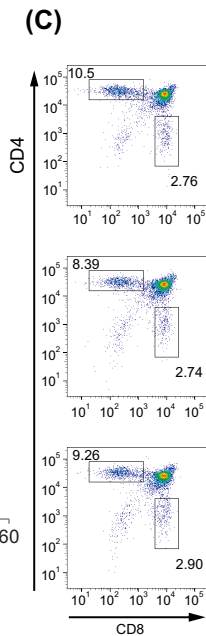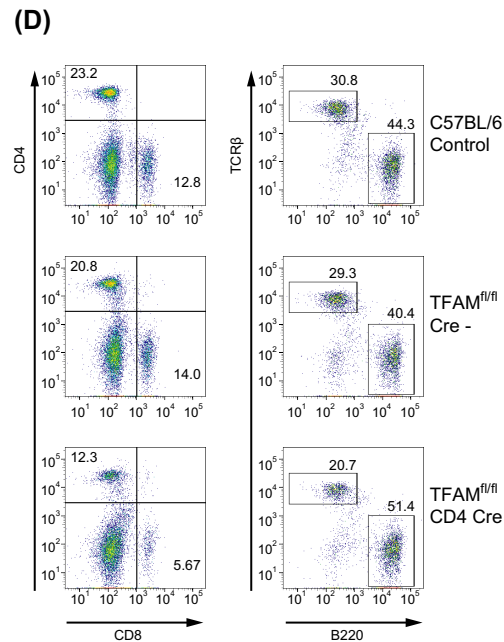

**(A)** TFAM<sup>fl/fl</sup> CD4Cre mice

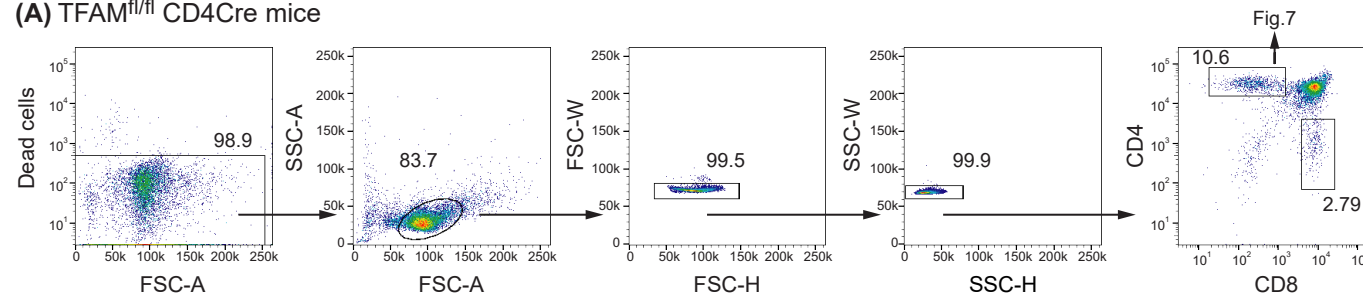

**(B)** Control mice

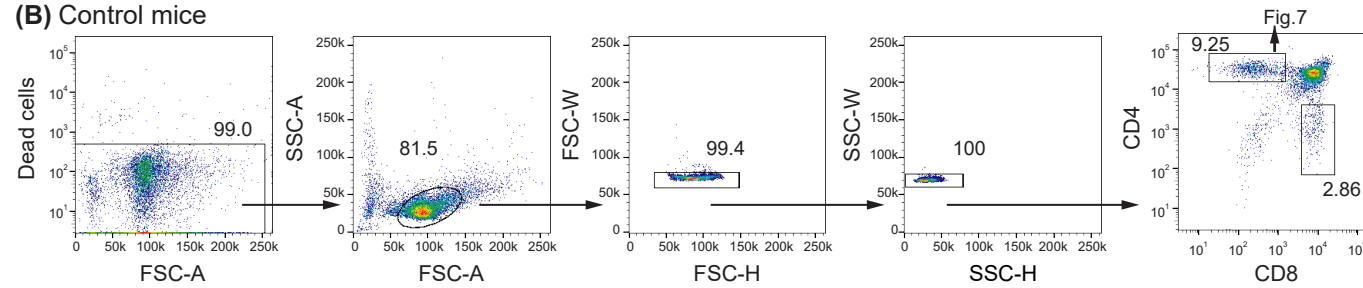

**(A) TFAM<sup>fl/fl</sup> CD4Cre mice**

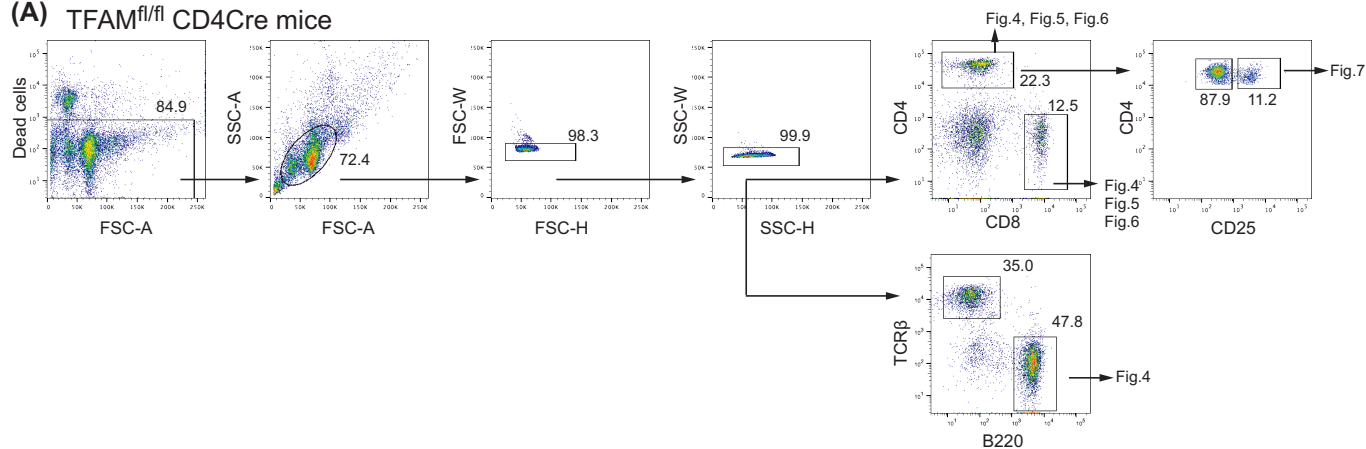

**(B) Control mice**

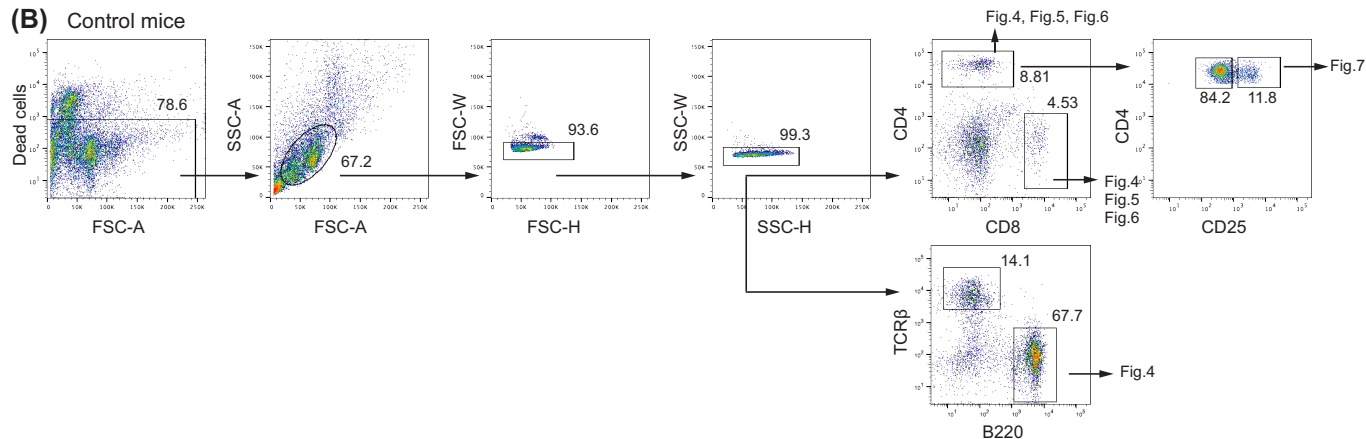

Supplement: Supplementary file 1 — Supporting Information Figure S1. Analysis of TFAM deletion in T cells. (A) T cells were prepared from the spleen of control and TFAMfl/fl CD4Cre mice. The gating strategy is presented in Figure 3. T cells were lysed, analyzed via SDS–PAGE, and immunoblotted with anti‐TFAM and anti‐β‐actin antibodies. (B) In the mitochondrial stress assay, changes in the oxygen consumption rate (OCR) were measured in real time after consecutive treatment with 1 μM of oligomycin, 2 µM FCCP, and 0.5 µM each of rotenone and antimycin A to induce mitochondrial stress. Mitochondrial activities of control and TFAM‐deficient CD4 T cells were analyzed using the Wave software (Seahorse Bioscience). (C) Flow cytometric plots showing CD4 and CD8 T‐cell population in thymocytes from control and TFAMfl/fl CD4Cre mice. (D) Flow cytometric plots showing CD4 and CD8 T cells, and B220+ and TCRβ+ cells from the spleen. Gating strategies are presented in Figures S2 and S3. Representative results from more than three independent experiments are shown. Figure S2. Analysis of TFAMfl/fl CD4Cre mice. Flow cytometric gating strategy used for the thymus. Figure S3. Analysis of TFAMfl/fl CD4Cre mice. Flow cytometric gating strategy used for the spleen. [file JIMR-2026-2594317-s001.pdf]
